# Age-dependence of sensorimotor and cerebral electroencephalographic asymmetry in rats subjected to unilateral cerebrovascular stroke

**DOI:** 10.1186/2040-7378-5-13

**Published:** 2013-11-19

**Authors:** Slavianka G Moyanova, Rumiana G Mitreva, Lidia V Kortenska, Ferdinando Nicoletti, Richard T Ngomba

**Affiliations:** 1 Institute of Neurobiology, Bulgarian Academy of Sciences, Acad. G. Bonchev Str. 23, 1113, Sofia, Bulgaria; 2 I.R.C.C.S., NEUROMED, Localita Camerelle, 86077, Pozzilli, (IS), Italy; 3 University Sapienza, Rome, Italy

**Keywords:** Stroke model, Aging, Middle-aged rats, Focal cerebral ischemia, Endothelin-1, Rat, Sensorimotor deficits, Electroencephalogram

## Abstract

**Background:**

The human population mostly affected by stroke is more than 65 years old. This study was designed to meet the recommendation that models of cerebral ischemia in aged animals are more relevant to the clinical setting than young animal models. Until now the majority of the pre-clinical studies examining age effects on stroke outcomes have used rats of old age. Considering the increasing incidence of stroke among younger than old human population, new translational approaches in animal models are needed to match the rejuvenation of stroke. A better knowledge of alterations in stroke outcomes in middle-aged rats has important preventive and management implications providing clues for future investigations on effects of various neuroprotective and neurorestorative drugs against cerebrovascular accidents that may occur before late senescence.

**Methods:**

We evaluated the impact of transient focal ischemia, induced by intracerebral unilateral infusion of endothelin-1 (Et-1) near the middle cerebral artery of conscious rats, on volume of brain damage and asymmetry in behavioral and electroencephalographic (EEG) output measures in middle-aged (11–12 month-old) rats.

**Results:**

We did not find any age-dependent difference in the volume of ischemic brain damage three days after Et-1 infusion. However, age was an important determinant of neurological and EEG outcomes after stroke. Middle-aged ischemic rats had more impaired somatosensory functions of the contralateral part of the body than young ischemic rats and thus, had greater left-right reflex/sensorimotor asymmetry. Interhemispheric EEG asymmetry was more evident in middle-aged than in young ischemic rats, and this could tentatively explain the behavioral asymmetry.

**Conclusions:**

With a multiparametric approach, we have validated the endothelin model of ischemia in middle-aged rats. The results provide clues for future studies on mechanisms underlying plasticity after brain damage and motivate investigations of novel neuroprotective strategies against cerebrovascular accidents that may occur before late senescence.

## Background

Age has been consistently identified as the strongest predictor of greater disability after ischemic stroke in humans [1]. The question of age-related differences in behavioral sensitivity to ischemia is important and has strong clinical relevance. Although stroke is mostly a disease of the elderly, the majority of experimental research is still conducted on young animals and, therefore, may not fully replicate the effects of ischemia on brain tissue in aged subjects [2-5]. In fact, epidemiological studies reveal that stroke occurs more often in old population (people aged ≥ 65 years) [1], thus for translational purposes, preclinical studies should be conducted on old animals [3]. However, studying stroke during the early phase of senescence, we may obtain clues for future investigations on the effects of neuroprotective and neurorestorative drugs for cerebrovascular accidents that may occur later in life. More importantly, there is a clear tendency nowadays for increasing incidence of stroke among human population aged 14–44 years [6]. The percent of this subpopulation has been increasing annually: while in 1983 this percentage was reported to be 3% for a group of age under 40 [7], in 1990 it increased to 8.5% for patients aged 15–45 years [8], and nowadays this percent is already 10-14% comprising patients of 18–45 years [References in 9]. The causes of this rejuvenation are multiple, the most important being the occurrence of co-morbidities in that age: smoking, diabetes mellitus, dyslipidemia, hypertension [9]. Elucidation of mechanisms of stroke and possibilities for recovery in younger than old patients, as well as revealing the impact of co-morbidities in these patients on stroke incidence may help finding new means for preventing stroke occurrence and new beneficial interventions. To help this task, new models of stroke in rodents in the middle range of the life span with or without co-morbidities are needed. It is certainly also feasible to induce consistent strokes in middle-aged or older animals even when they are also diabetic and hypertensive [10].

Infusion of Et-1 near the middle cerebral artery (MCA) produces cerebral ischemia and brain damage [11]). We found previously that transient focal ischemia induced by unilateral infusion of Et-1 (60 pmol) adjacent to MCA in conscious young adult rats (3-4-months old) resulted in moderate neuropathological outcome in the cortex and striatum, marked disturbances in the contralateral part of the body in the postural hang reflex (PHR) test, and in the limb placing (LP) test, as well as moderate EEG changes in the forelimb region of the ipsilateral somatosensory cortex up to 14 days after the Et-1 infusion [12-14]. To date, a number of studies have shown that the neurological outcome of ischemia is more severe in old rats (2 years of age) than in young adult rats (References in [2,4,5]). However, to our knowledge there have been only a few studies examining the outcome of cerebral transient ischemia in middle-aged rodents [15-18]. Therefore, we attempted to produce brain infarcts in middle-aged rats, but with a larger dose of Et-1 (150 pmol), in order to: (1) determine the size of brain infarcts after unilateral Et-1 infusion near the MCA in middle-aged rats and to compare it to that in young rats; (2) determine the ischemia-induced asymmetry of sensory-motor and reflex functions in middle-aged rats in comparison with that in young rats; and (3) examine the hemispheric asymmetry in the EEG in both groups subjected to ischemia.

## Methods

### Animals, surgery, and ischemia method

All experiments were carried out in accordance with the National Institute of Health Guide for the Care and Use of Laboratory Animals (NIH Publications No. 80–23) revised 1996 or the UK Animals (Scientific Procedures) Act 1986 and associated guidelines, or the European Communities Council Directive of 24 November 1986 (86/609/EEC). Formal approval for conducting the experiments was obtained by the Ethics Committee of the Institute of Neurobiology (Sofia, Bulgaria) and the Ethics Committee of the Neuromed Institute (Pozzilli, Italy). All efforts were made to minimize the number of animals used and their suffering.

Male Wistar rats were used for the study: n = 38 young adults aged 3-4-months and weighing 240–280 g b.w. (group Y), and n = 34 middle-aged adults aged 11–12 months and weighing 350–400 g, b.w. (group M). According to Sengupta’s correlation of human years with rat days during the entire life span [19], the 12-month-old middle-aged rats used in this study correspond to human age of about 27 years. This is in the range of 18–45 years of human subpopulation (10-14%) reported to have received a stroke [References in 9]. The surgery procedure used has been described previously [14,20]. Briefly, the animals were housed before surgery 4–5 to a cage in standard laboratory conditions on a 12 h light/12 h dark cycle, with access to food and water ad libitum. The rats were anesthetized with a mixture of ketamine (Calypsol, Gideon Richter, Budapest, 70 mg/kg) and Xylazine (Alfasan-Woerden, Holland, 4 mg/kg) injected intramuscularly and then fixed in a stereotaxic apparatus (Narishige Sci Inst., labs, Japan). EEG electrodes were implanted into homologous points of both hemispheres in three cortical areas at coordinates (Bregma as reference) as follows [21]: forelimb region of the somatosensory cortex, S1FL at antero-posterior, AP = + 1.2 mm and lateral-medial, LM = ± 4.0-4.5 mm; barrel field of the somatosensory cortex, S1BF at AP = -0.3 mm, LM = 6.0-6.5 mm and primary visual cortex, V1 at AP = -7.0 mm, LM = 4.5 mm. For EEG recordings in S1FL and V1, we used small stainless steel screw electrodes (MCSM 1x2 mm; AgnTho’s AB, Sweden) with pieces of stainless steel wire coated with teflon and soldered to their caps. For epidural EEG recordings in S1FB, we used stainless steel wire coated with Teflon (A-M systems Inc, USA) with bare diameter of 127 microns and inserted bilaterally into the pre-made holes in the calvarium. Two other pieces of wires were inserted bilaterally into the dorsal neck muscles for recording of muscle activity (EMG) and movements. Two miniature stainless steel screws, one fixed on the skull above frontal bone and the other, posterior to lambda, were used for common reference and ground, respectively. Two additional screws were fixed onto the skull for anchoring the implant. All wires were connected to pins of a female miniconnector.

A 21-gauge stainless steel guide cannula was implanted into the left hemisphere (for Et-1 infusion performed two weeks later) at coordinates for the young animals as follows: AP = + 0.2 mm, LM = 5.5 mm, and DV = - 4.1 mm below the dura. For the middle-aged rats, LM and DV coordinates were corrected according to body weight [21]. Because of variations in skull thickness between rats of different age, the target dorsal-ventral coordinate was obtained in both groups of animals using as a reference the point at the surface where a hole will be made for insertion of the guide cannula, instead of the point over the cranium at Bregma. A mound of self-curing Duracryl resin (Spofa Dental, Prague, Czech Republic) was built up around the guide cannula and all electrodes to secure them onto the skull together with the female connector.

Stroke was induced about two weeks after the surgery by means of unilateral infusion of 150 pmol endothelin-1 (Et-1) in sterile saline (3 μl) into the left hemisphere by means of a Hamilton syringe connected with a short length of polyethylene tube to a 27-gauge injection cannula introduced into the guide cannula and protruding 2 mm below its tip. The Et-1 solution (or saline) was infused slowly (1 μl/1 min) for 3 min. During the infusion, the already-habituated rat was maintained on a temperature feedback-controlled pad (Digiterm, Yukon-PC, Sofia, Bulgaria).

Prior to Et-1 infusion, all rats were tested for symmetrical limb extension when held in the air by the tail. They were then allocated to ischemia or sham groups (separately for the Y and M groups) in a counterbalanced fashion depending on whether they were to be infused with Et-1 (n = 29 ischemic young rats, Y_i_, n = 27 ischemic middle-aged rats, M_i_), with only its vehicle, i.e. saline in a volume of 3 μl or not infused but having implanted cannula (n = 9 sham young rats, Y_s_ and n = 7 sham middle-aged rats, M_s_). The nonischemic rats that did or did not receive intracerebral infusion of saline were combined into one control group (sham, separately for Y and M animals) because they did not differ in all behavioral tests. Ischemic and sham rats were randomly assigned to be killed at 3 days post Et-1 for the infarct volume measurements or at 14 days for behavioral and EEG recordings. A separate group of five rats was used in a longitudinal study of evolution of EEG in the somatosensory cortex of both hemispheres. These rats were infused with Et-1 unilaterally near the MCA when they were young adults (3-4-months old) and then EEG was recorded for 8 months starting from the first month after the Et-1 administration. By the end of the experiment, the rats were 11-12-month-old, i.e., middle-aged.

### Behavioral tests

Animals were handled every day for 1 week before the onset of experiments. Two experimentators carried out all behavioral testing were blinded to the type of cerebral infusion (Et-1 or saline). Behavioral procedures were conducted between 10:00 a.m. and 14:00 p.m. Four behavioral tests were performed: posture/hang reflex (PHR), limb placing (LP), limb use asymmetry in postural support in cylinder (LUA), and adhesive removal (ATR). All these tests are accepted as important tests for long-term assessment after stroke to improve translation from bench to bedside [22]. They have been described in detail previously [13,14,20]. The tests were performed before Et-1 or saline (T0) and then at 1 hour (H1), 4 hours (H4), 1 day (24 hours, D1), 3 days (D3), 7 days (D7), and 14 days (D14) after Et-1 or saline. For the T0 session, mean of three observations during three consecutive days was obtained. The PHR test (described below) performed 10–15 min after the Et-1 infusion was used as an inclusion criterion for rats having undergone stroke [23]. We examined the asymmetry between behavioral performances of left (ipsilateral) and right (contralateral) body sides.

### Posture/reflex hang (PHR) test

The PHR test measures upper body posture [24]. The rat was held by its tail 50 cm above a platform. A rat that extended both forelimbs toward the platform received a score of 3 (normal); a rat that flexed the right forelimb (contralateral to the damaged hemisphere) received a score of 2 (mild neurological deficit). Then the rat was put on a platform and a lateral push was applied behind the shoulders. If a rat with a score of 2 had decreased resistance to pressure towards the right side of the body and slid the right limbs, it received a score of 1 (moderate neurological deficit); a score of 0 (severe neurological deficit) was given to a rat that had right forelimb flexion, curled its thorax toward the tail when suspended (torso twisting), had decreased resistance towards the right side of the body, circled towards the right side, or had loss of walking and righting reflex when on the table. The asymmetry was assessed by subtracting the contralateral scores from the ipsilateral ones.

### Limb placing (LP) test

The LP test was performed on hand-held rats in order to examine placing of forelimbs and hindlimbs in response to visual, vibrissae, tactile, and proprioceptive stimulation [25]. The scores were as follows: score 2, the rat performed immediate and correct placing on the platform (normal); score 1, the rat performed with a delay (>/2 s) and/or incompletely (mild or moderate deficit); and score 0, the rat did not perform normally (severe deficit). Summing the scores of all eight LP subtests yielded a total maximal neurological score of 16 in a normal rat for each side [13]. A lower total score implicated impairment in sensorimotor integration contralateral to the ischemic hemisphere (right limbs). The asymmetry in performance was assessed by subtracting the contralateral scores from the ipsilateral ones.

### Limb-use asymmetry (LUA) test

The Schallert LUA (cylinder) test was used to assess forelimb use for postural weight support during exploratory activity [26] within a transparent 20 cm diameter/30 cm height cylinder. The first forelimb to touch the wall was scored as an independent placement for that forelimb. If both forelimbs were simultaneously placed against the wall during rearing, this was scored as both. Scores were obtained from a total number of 20 full rears in each session. Percentage was calculated for ipsilateral forelimb use and for contralateral forelimb use relative to the total number of uses (ipsilateral + contralateral + simultaneous) for each rat at each session. Then the asymmetry was calculated by subtracting the percentage use of the contralateral (right) forelimb from the percentage use of the ipsilateral (left) forelimb: a higher asymmetry index value indicated an increase of reliance on the ipsilateral forelimb and a decrease of use of the contralateral forelimb.

### Adhesive tape removal (ATR) test

The bilateral ATR test [26] was used to measure forelimb somatosensory asymmetry. Rectangular (1-cm^2^) adhesive tapes were placed with equal pressure on the dorsal side of the wrists of both forepaws. The order of placing of the adhesive (right or left) was alternated each day and randomized within each group. The rats were trained five times a day for 3 days before Et-1 infusion to obtain a stable basal level of performance (removal time 20–30 s). Then, each animal underwent four trials (with a 1–2 min inter-trial interval) on pre- and all post-Et-1 sessions. The mean time (latency) and the order of removing of tapes from the ipsilateral and contralateral forepaws were recorded. A trial ended when the rat either removed both adhesive tapes or 180 seconds elapsed. The asymmetry in the time of removal of the adhesive tape was obtained by subtracting the latency to remove the tape with the contralateral forepaw from the latency to remove the tape with the ipsilateral forepaw. As a measure of asymmetry, we also used the percentage of first removal of the adhesive tape by the ipsilateral forepaw of total number of removals. We did not perform ATR test at H1 and H4 because this test was shown to reliably detect chronic sensorimotor deficits usually beginning from the first day after the induction of stroke [27].

### EEG recording

EEG recordings began 7–10 days after surgery and were performed on free-moving rats. The rat was placed in a recording cage (Faraday box) equipped with a rotating mercury swivel (commutator) mounted on the box’s ceiling, which allowed the rat to move around in the box. The outputs of this commutator were fed to amplifiers of an EEG machine (Nihon Kohden, Tokyo, Japan) with a high-pass filter at 0.16 Hz (HF, -3 dB), a low-pass filter at 70 Hz (LF, -3 dB), and a notch filter at 50 Hz (for the power line noise reducing). In addition to EEG, electromyogram (EMG, time constant TC =0.1 s, LF at 500 Hz) and movements (TC = 1.0 s, LF at 15 Hz) from the dorsal neck muscles were recorded to help extraction of suitable EEG epochs for further processing without any artifacts due to movements. EEG recordings of at least 15 min duration were made during each session. For the background (T0) session, we made three recordings on three consecutive days before Et-1. Then, EEG recordings were performed at 15 min (M15), 1.25 hours (H1), 4.25 hours (H4), 24 hours or day 1 (D1), and on day 3 (D3), day 7 (D7), and day 14 (D14) after Et-1. The EEG recordings for each rat were made after the behavioral assessment of the rat.

### EEG analysis

Taking into account the non-stationary nature of the EEG signals, for the spectral EEG analysis short epochs of 4-s duration were chosen from the raw EEG, which can be considered stationary under constant behavioral conditions of the animal (quiet awaking state, immobility with head up and eyes open). The epoch length of 4-s is commonly used in the EEG analysis [28]. Using Welch’s periodogram method, power densities (pd) were estimated by means of Hanning windowing and standard fast Fourier Transform (FFT) algorithm (program 1 t of the BMDP Statistical Software, Los Angeles, 1990). The spectra contained 125 discrete frequencies (bins) at each 0.25 Hz in frequency range from 1 to 32 Hz. Then mean power spectra of 16 to 25 epochs (mean number n =20) of EEG was obtained and Fourier coefficients for each frequency bin (spectral power densities) were averaged for all epochs in a session, for each cortical deviation, each time session, and each rat. Subsequently, the EEG spectral profiles were averaged for all rats in each group and plotted for each time session before and after the induction of stroke. Then, power density brain symmetry index (pdBSI) was calculated as a measure of asymmetry in EEG recorded along homologous channel pairs of the two hemispheres [29]. The pdBSI was defined as the mean of the absolute values of the difference in mean hemispheric power in the frequency range of 1–32 Hz for each time session Ti (T0, M15, H1, H4, D1, D3, D7, D14) as follows:

$$\mathrm{pdBSI}\left(\mathrm{Ti}\right)=1/125\sum_{j=1}^{125}|[\mathrm{Cj}\left(\mathrm{Ti}\right)-\mathrm{Ij}\left(\mathrm{Ti}\right)]/\left[\mathrm{Cj}\left(\mathrm{Ti}\right)+\mathrm{Ij}\left(\mathrm{Ti}\right)\right]|$$

where:

*C*_*j*_*(T*_*i*_*) = 1/3 [a*_*j*_^*2*^*(FC) + a*_*j*_^*2*^*(BC) + a*_*j*_^*2*^*(VC)]* for the right contralateral hemisphere (C)

*I*_*j*_*(T*_*i*_*) = 1/3 [a*_*j*_^*2*^*(FI) + a*_*j*_^*2*^*(BI) + a*_*j*_^*2*^*(VI)]* for the left ipsilateral hemisphere (I),

F indicates the forelimb region of the somatosensory cortex (S1FL); B indicates the barrel field of the somatosensory cortex (S1BF); and V indicates the primary visual cortex (V1). Here, a_j_ are the Fourier coefficients (pd) at each of total of 125 frequency bins in the FFT power spectra from 1 to 32 Hz. Units of pdBSI ranged from 0 (no asymmetry) to 1 (maximal asymmetry). Additionally, in rats examined longitudinally, we measured number and duration of high-voltage spindles (HVS) in the S1BF cortical areas of both hemispheres 3–4 times per month during 8 months after the induction of stroke. For this purpose, cumulative 10-min immobility periods were collected off-line from periods of quit waking state when the rat was standing quiet in the recording box with open eyes without EEG and EMG signs of movements, drowsiness or sleep (EMG recording from dorsal neck muscles). The HVS was considered as such if it met the criteria described by Buzsaki et al [30]. No motor manifestations that looked convulsive were observed concomitantly with the neocortical HVSs. Two parameters of HVS were measured from the EEG records, HVS incidence (number of HVS in 10-min cumulative waking-immobility period) and duration (in s) of each HVS burst.

### Quantification of ischemic damage

For quantification of ischemic damage, three days after the induction of stroke the brains of 11 young rats and 11 middle-aged rats infused intracerebrally with either Et-1 or saline were removed and sliced coronally at 2 mm intervals. Three days were chosen as time point for estimation of ischemia-induced brain damage, because it had been shown previously that the damage was fully matured by 3–7 days post-Et-1 [11]. The slices were placed in 2% 2,3,5-triphenyltetrazolium chloride (TTC) (Sigma Chemical Co.) in saline for 20 min at 37°C. The posterior surface of each stained slice were scanned with an HP Scanjet 4370 and the areas lacking TTC stain (infarcted) were demarcated and measured (AutoCAD2000). Volume of injury was calculated as the sum of the area from each section, multiplied by the distance between sections. The infarct volume expressed as a proportion of contralateral hemispheric volume to correct for differences in brain size resulting from edema.

### Statistics

All data were verified for normality and homogeneity of variance and then analyzed using Sigma Plot 11.0 (Systat Software Inc), Statistica 7.0 (Statsoft, Tulsa, OK, USA) and Microsoft Excel (Microsoft Office 2010, Microsoft Corp.). Since in most cases the data did not fulfill the criteria for normality, or were measured in scores, nonparametric Friedman ANOVA and Wilcoxon matched pairs test were used for comparisons between post- and pre-Et-1 values and between ipsilateral and contralateral values. Mann–Whitney rank U-test was used for comparisons between the two ischemic age groups and also between the ischemic and sham corresponding groups. For all statistical analyses, a P value of less than 0.05 was deemed significant.

## Results

### Mortality, exclusion of non-ischemic animals, and weight

Most of the rats of both age groups infused with Et-1 showed well-expressed neurological deficits as soon as 10–15 min following the infusion. The percentages of excluded rats (infused with Et-1 but showing no early signs of stroke) in both age groups did not differ significantly: 6.8% (2/29) in the Y_i_ group and 11.1% (3/27) in the M_i_ group (P > 0.05, χ^2^ test). The difference in the mortality rate (within 24 h after stroke) between Y_i_ and M_i_ rats remaining after exclusion was also not statistically significant: Y_i_ = 3.7% (1/27), M_i_ = 8.3%, (2/24), P > 0.05, χ^2^ test.

**Figure 1:**
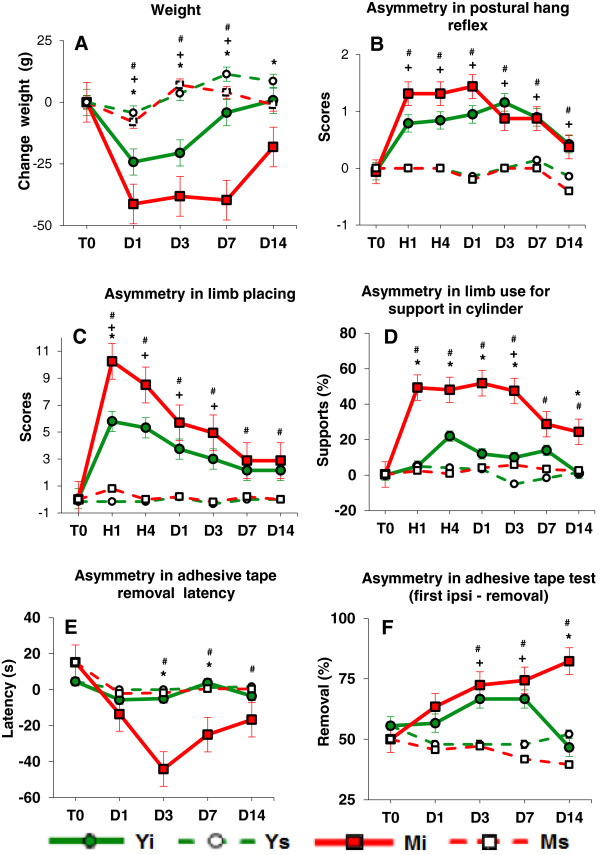
**Age-related differences in body weight and asymmetry in posture/sensorimotor performance after unilateral Et-1-induced occlusion of MCA in conscious rats. A**: Changes in body weight versus pre-Et-1 weight. The ischemic rats (Y_i_, n=26 and M_i_, n=22) loose more weight compared with the sham rats (Y_s,_ n=9 and M_s,_ n=7), and the M_i_ rats loose more weight than the Y_i_ rats. **B**: PHR test. The asymmetry in Y_i_ and M_i_ rats was greater (the contralateral scores are smaller than the ipsilateral ones) than that in Y_s_ and M_s_ rats. No difference between the asymmetry in both ischemic age groups was found. **C**: LP test. Asymmetry in Y_i_ and M_i_ animals was greater than that in Y_s_ and M_s,_ correspondingly. The M_i_ rats showed greater asymmetry than the Y_i_ rats during the acute phase. **D**: LUA test. The difference in asymmetry between M_i_ and Y_i_ rats was significant during the whole experiment. **E**: ATR test - latency. The difference between the M_i_ and Y_i_ rats was significant only during the chronic phase of ischemia. **F**: ATR test - first removal of the adhesive tape from the ipsilateral forepaw (number in % from all removals). The Yi rats recovered at D14, while the M_i_ rats showed an increase in the asymmetry by that time. On the abscissa: T0- before Et-1 or saline, H1 - 1 hour, H4 - 4 hour, D1 - one day, D3 - 3 days, D7 - 7 days, and D14 - 14 days after Et-1 or saline. Means ± SEM are presented. Values at T0 were calculated for all Y rats (Y_i_ and Y_s,_ n = 35) and for all M rats (M_i_ and M_s,_ n = 29). Significant difference at P<0.05 (Mann-Whitney U): + Y_i_ vs. Y_s,_ # M_i_ vs. M_s,_ * Y_i_ vs. M_i_.

After stroke, the remaining rats ate less and their body weight declined progressively; however, these changes were transient, peaking 1–7 days after Et-1-induced stroke, with a maximum mean loss of 10.3% for M_i_ rats and 8.1% for Y_i_ rats (Figure 1A). The decline in body weight was greater in M_i_ than in Y_i_ rats. An increase in weight and complete recovery to pre-ischemia weight was observed by D14 in both Y_i_ and M_i_ rats.

### Quantification of infarct volume

Measurement of the infarct volume using TTC staining revealed no significant difference in the volume of brain infarcts between young and aged ischemic rats (37.0 ± 5.1% for Y_i_ rats and 41.0 ± 3.9% for M_i_ rats).

### Behavioral tests

Before Et-1 (at T0, Figure 1B,C,D,E,F), no significant difference in performance of the two age groups of animals was noted; there was also no significant age-dependent change in sham, (non-ischemic) rats in the four behavioral tests. Sham rats had no sensorimotor deficits across time sessions. Following Et-1-induced stroke, the inter-subject variability of parameters measured in four posture/reflex and sensorimotor tests was greater in the M_i_ group than in the Y_i_ group.

### Posture/reflex hang (PHR) test

The PHR test was highly sensitive to Et-1-induced ischemia in both age groups, but no age-related difference was observed. In the acute phase of ischemia the majority of rats of both age groups showed a right posturing of the body, lack of resistance to lateral left push, and flexion of the contralateral (right) forelimb with characteristic clinching of this forelimb towards the breast, a posture often seen in people with stroke. An asymmetry in the performance of the left limbs/body (unimpaired) and that of the right limbs/body (impaired) was observed with the PHR test in all ischemic rats during the whole period of observation (Figure 1B). The asymmetry in Y_i_ and M_i_ was significantly greater than in the respective sham groups Y_s_ and M_s_ up to 14 days after the insult (Friedman ANOVA, Time effect, P < 0.001 and Wilcoxon post-hoc tests). However, there was a clear tendency for the M_i_ rats to exhibit more severe onset and more expressed asymmetry than the Y_i_ rats. The rate of recovery of the asymmetry in the PHR test was identical in both groups.

### Limb placing (LP) test

Both young and middle-aged ischemic rats were impaired in the contralateral (right) LP performance: for Y_i_ rats from H1 to D3 and for M_i_ rats from H1 to D14, compared with the corresponding sham rats (Mann–Whitney U-test) (Figure 1C). A time effect (Friedman ANOVA, Chi Sqr. = 93.7; P < 0.001) was present, indicating a deficit of contralateral forelimb and hind limb placing in all ischemic rats at all time sessions compared with the pre-Et-1 (at T0) scores (Wilcoxon matched pairs test), with no full recovery until D14. M_i_ rats exhibited an earlier onset of deficits than Y_i_ rats (significant difference at H1). The asymmetry in M_i_ rats was greater than in Y_i_ rats because the deficits of the contralateral limbs in M_i_ rats were more expressed than in Y_i_ rats. The asymmetry in the Y_i_ rats was transient and reached the level of asymmetry shown by sham rats on days 7–14 after Et-1 infusion, while the asymmetry in the M_i_ rats recovered later than in Y_i_ rats.

### Limb-use asymmetry (LUA) test

Before Et-1 infusion, no rats showed asymmetrical use of the forelimbs during postural support in the LUA (cylinder) test. M_i_ rats showed significant increase in asymmetry both in the acute and in the chronic phases of ischemia as compared to both the pre-Et-1 baseline performance (at T0) and the asymmetry measured in the M_s_ group (Figure 1D). This asymmetry was due to a significant reduction in the use of the impaired contralateral (right) forelimb and increased reliance on the ipsilateral (left) forelimb for postural support. Ignoring the dynamics in time, the mean value of asymmetry in postural support for the entire period of post-stroke observation (all 6 time sessions) was 41.67 ± 4.85% (mean ± SEM) for M_i_ rats and only 12.9 ± 3.0% for Y_i_ rats.

### Adhesive tape removal (ATR) test

Before Et-1 infusion, there was no significant difference between ischemic and non-ischemic rats of both age groups or between latency to remove the adhesive tape from both forepaws. In M_i_ rats, the latency to remove the tape from the contralateral forepaw was much greater than that measured in Y_i_ rats and thus, the difference in the asymmetry in latency in M_i_ and Y_i_ rats reached statistical significance at D3 and D7 (Figure 1E).

Both groups showed symmetrical preference in the first removal of the adhesive tape before Et-1 infusion, estimated to be about 50% (Figure 1F). The preference of first removal of the adhesive from the unimpaired ipsilateral forelimb increased with time after ischemia in rats of both age groups, indicating that the use of the contralateral forepaw to perform this task was severely affected by ischemia. However, comparisons with the corresponding sham rats with the Mann–Whitney U-test demonstrated that Y_i_ rats showed full functional recovery at the 14th day after Et-1 infusion, while M_i_ rats did not show any recovery across the observation period until D14.

### EEG analysis

**Figure 2:**
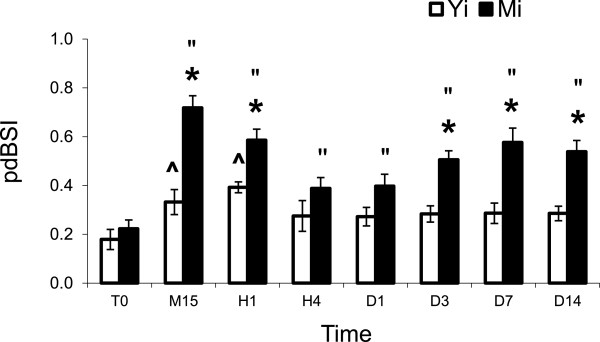
**EEG power spectral density derived Brain Asymmetry Index (pdBSI).** The difference between pdBSI estimated in young ischemic (Y_i_, n = 7) and middle-aged ischemic (M_i_, n = 7) rats was significant during the acute phase (at M15, H1) and during the chronic phase of ischemia (at D3, D7, D14). Mean ± SEM. * Y_i_ vs. M_i_ (Mann–Whitney test); **^** Y_i_ vs Y_T0_; “ M_i_ vs. M_T0_ (Wilcoxon matched pairs test). Time sessions are as in Figure 1. Significant difference at P < 0.05.

We measured the EEG asymmetry between homologous channels for three cortical regions (S1FL, S1BF, and VI) in the ipsilateral left (ischemic) and contralateral right (non-ischemic) hemispheres in ischemic young and middle-aged rats by means of pdBSI. Figure 2 illustrates the evolution of pdBSI after Et-1-induced ischemia. No significant difference was found in the baseline pdBSI (before Et-1, T0) between the two groups of rats. Nonparametric Friedman ANOVA demonstrated a significant effect of factor Time in Y_i_ rats (Chi Sqr. = 14.9, P = 0.038) and M_i_ rats (Chi Sqr. = 30.7, P = 0.00007). In Y_i_ rats, the increase in EEG asymmetry compared with the pre-ischemia values was significant at M15 and H1, while in M_i_ rats, EEG asymmetry increased significantly at all time sessions (Wilcoxon matched pairs test). The difference between Y_i_ and M_i_ rats was significant at M15, H1, D3, D7, and D14 (Mann–Whitney U-test).

**Figure 3:**
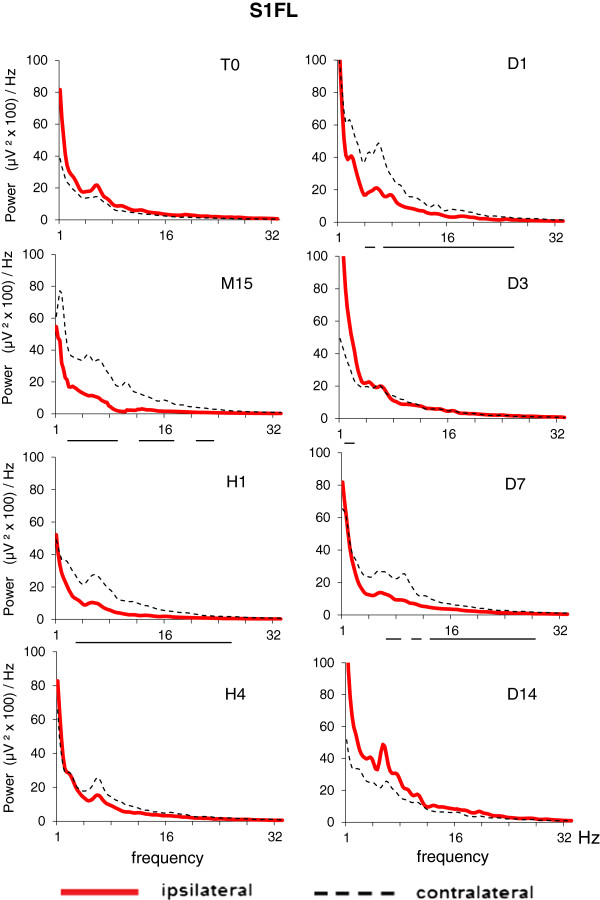
**EEG spectral density profiles in ipsilateral (left) and contralateral (right) homologous points of somatosensory cortex, forelimb region (S1FL) before (T0) and after Et-1-induced stroke in conscious middle-aged rats (M**_**i**_**, n = 14).** The time sessions are as in Figure 1. The horizontal lines below pairs of ipsi/contralateral spectral profiles for each time session denote statistical difference at each frequency bin (0.25 Hz) from 0 to 32 Hz (Wilcoxon matched pairs test with P < 0.05).

**Figure 4:**
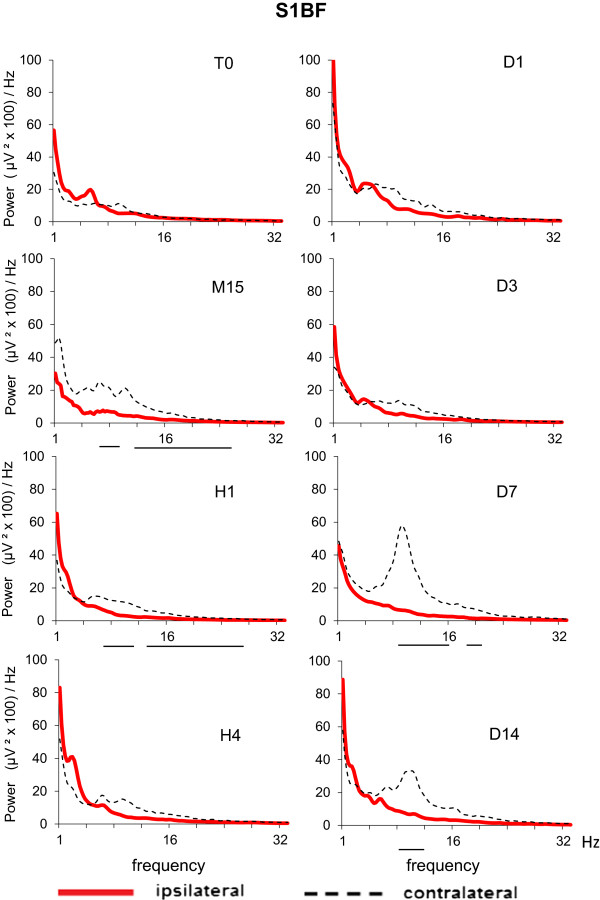
**EEG spectral density profiles in ipsilateral (left) and contralateral (right) homologous points of somatosensory cortex, barrel field (S1BF) before (T0) and after Et-1-induced stroke in conscious middle-aged rats (M**_**i**_**, n = 14).** The time sessions are as in Figure 1. The horizontal lines below pairs of ipsi/contralateral spectral profiles for each time session denote statistical difference at each frequency bin (0.25 Hz) from 0 to 32 Hz (Wilcoxon matched pairs test with P < 0.05).

**Figure 5:**
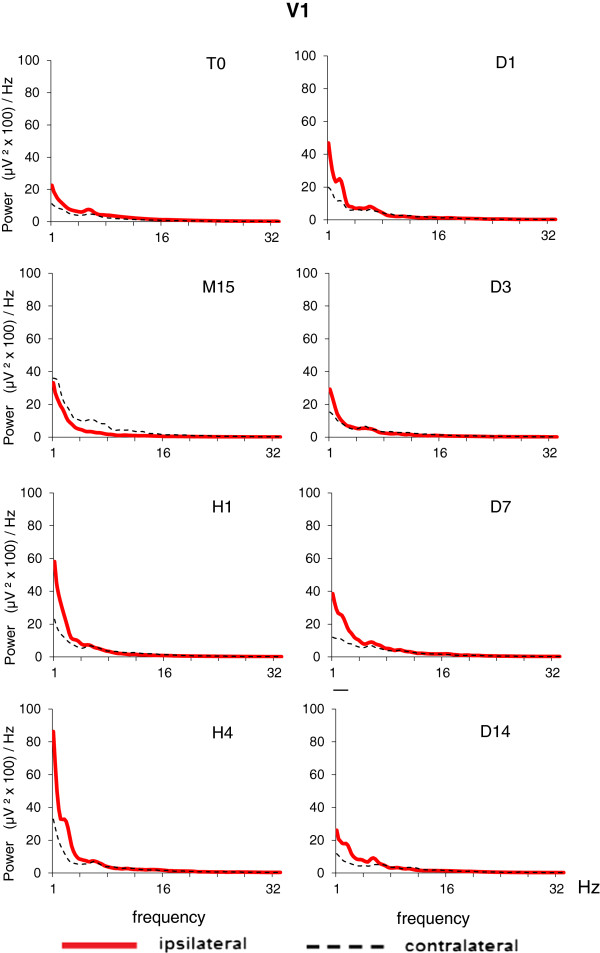
**EEG spectral density profiles in ipsilateral (left) and contralateral (right) homologous points of primary visual cortex (V1) before (T0) and after Et-1-induced stroke in conscious middle-aged rats (M**_**i**_**, n = 14).** The time sessions are as in Figure 1. No statistical difference was found between power density values derived from ipsilateral and contralateral homologous points of the visual cortex.

**Figure 6:**
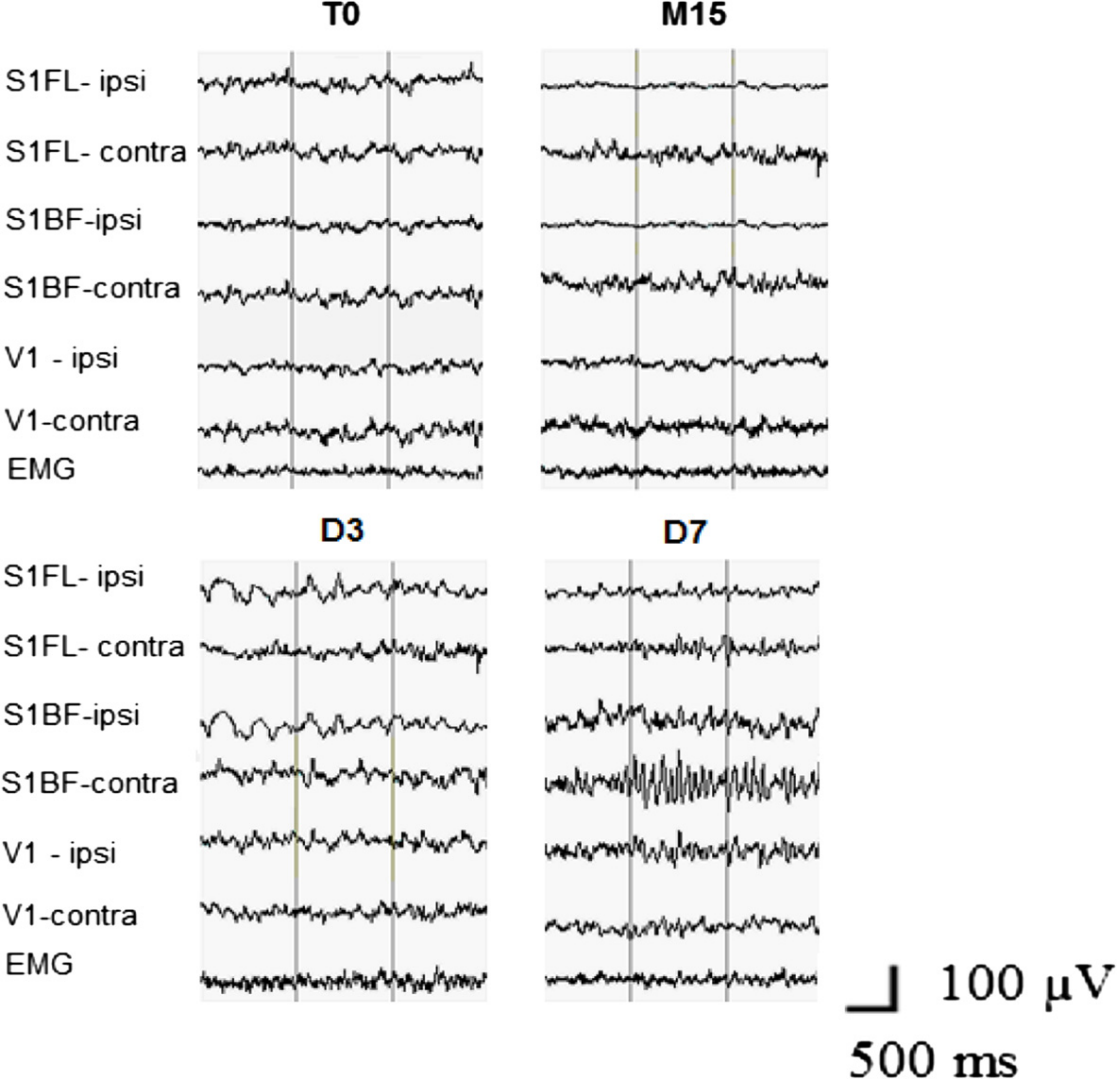
**Representative EEG segments are shown from the homologous areas of S1FL, S1BF, V1 in ipsilateral and contralateral hemispheres, together with electromyography record (EMG) in a middle-aged rat before (T0) and then at M15, D3, and D7 after Et-1.** Note the appearance of HVS in the contralateral S1BF at D7 with frequency of waves corresponding to the peak at 9.5 Hz in the spectral profile shown in Figure 4 at D7.

In order to examine the regional specificity of EEG after Et-1 infusion in middle-aged rats, we constructed EEG spectral density profiles for each of the three cortical areas in the ipsilateral and contralateral hemispheres. A significant difference between the EEG profiles in ischemic and nonischemic hemispheres occurred in S1FL (Figure 3) and S1BF (Figure 4); the difference in V1 was negligible (Figure 5). During the acute ischemic period (M15 and H1), the difference between ipsilateral and contralateral spectral profiles (Wilcoxon matched pairs test at each frequency bin of the spectral profiles) in both somatosensory areas was due to a reduction in the EEG power in the ipsilateral ischemic hemisphere below that at T0 and to an increase in the EEG power in the contralateral nonischemic hemisphere above that at T0. At H4, an increase in slow delta waves above that at T0 was evident from the spectral profile in ipsilateral S1BF. The next day (24 hours after Et-1 infusion, D1), we found an augmentation of the EEG power in both, ipsilateral and contralateral S1FL areas compared with that before the ischemia with statistically significant differences between them in all frequency bands except the slowest. Later (D3), there was an increase in the power of the slow-frequency waves (delta band) in the ipsilateral ischemic S1FL above that in the contralateral homotopic area (Figure 3). In the contralateral nonischemic S1BF, an increase in the power of in the alpha range (8–12 Hz) with peak at 9.5 Hz was prominent at D7 and D14 (Figure 4). This peak corresponded to the frequency of the spontaneous EEG high-voltage spindles (HVS) in this area (see the raw EEG in S1BF at D7 in Figure 6). Sharp waves, spikes or spike-wave discharges which are characteristic for the epileptic EEG had not appeared in both age groups.

**Figure 7:**
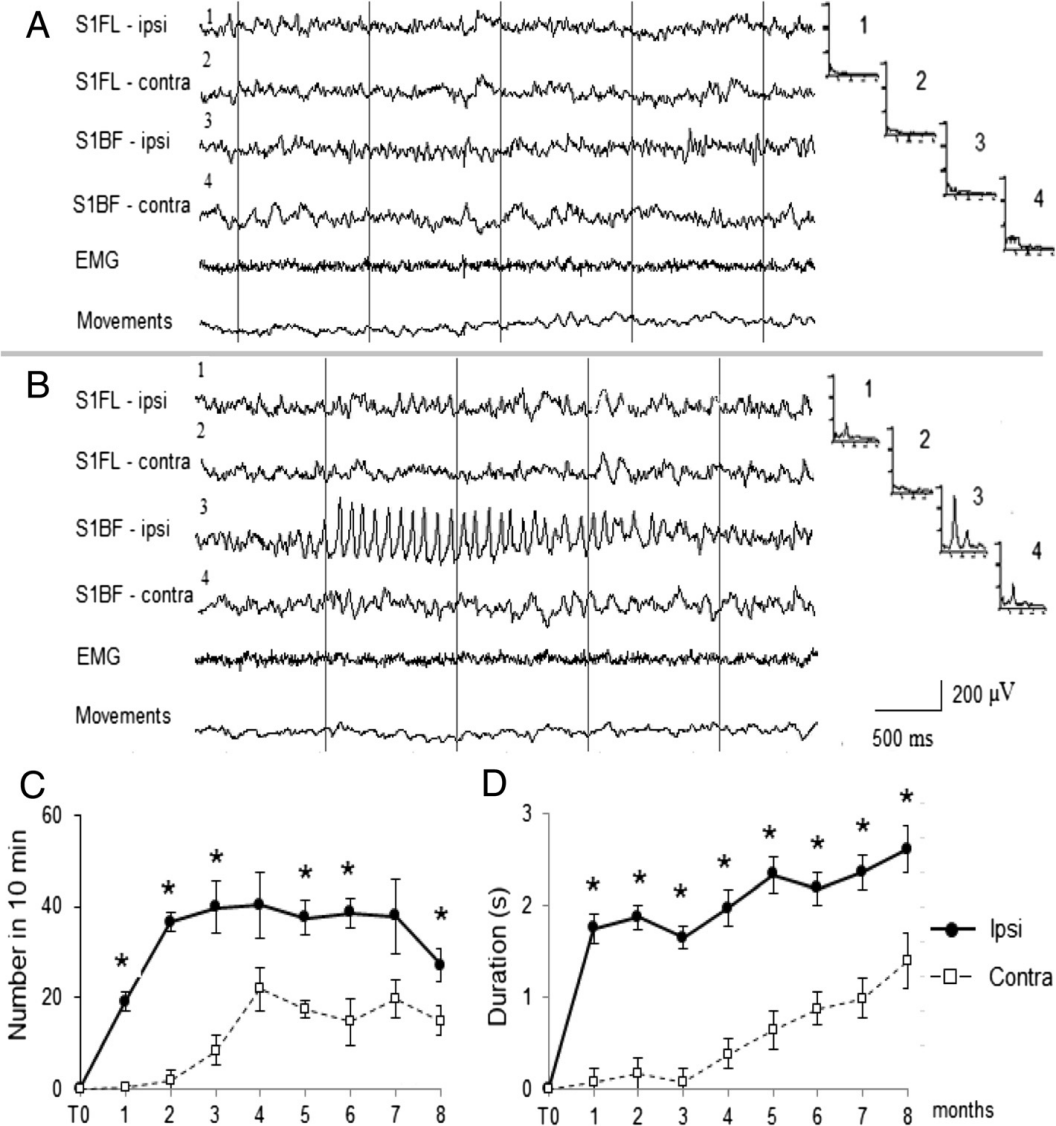
**Example of EEG evolution derived from a rat studied longitudinally from month one (the rat is 4-month-old) to month eight (the rats is 12-month old) after Et-1. A**: background EEG raw record before Et-1. **B**: 5 months after Et-1. The EEG records are shown in homologous (ipsi- and contralateral) areas of S1FL and S1BF. High-voltage spindles (HVS) are seen in the ipsilateral S1BF. The recording of electromiography (EMG) and rat’s movements show that the rat is immobile during the HVS. Corresponding mean spectral FFT profiles (n = 20 EEG epochs) are shown on the right of each EEG channel. On the abscissa: frequency from 1 to 32 Hz, on the ordinate: EEG spectral power, μV^2^/Hz. Note the large peak at 9.5 Hz in the spectral FFT profile (B3) corresponding to the frequency of the HVS waves in the ipsilateral S1BF and absence of such a pick in the contralateral S1BF spectral profile (B4). Number and duration of the HVSs (in 10-min epochs of waking immobility) in the ipsilateral and contralateral S1BF are shown in **C** (number) and **D** (duration) during 8-months follow-up. T0 – before Et-1. Data values are means ± S.E.M. for 10 min EEG epochs. * P <0.05 (Wilcoxon nonparametric test).

In rats of the longitudinal group in which we had EEG recordings from one to eight months after the Et-1 infusion, we observed HVSs in S1BF, which were expressed much more (in number and duration) ipsilaterally than contralaterally during the entire period of recording (Figure 7). HVSs appeared rarely in the contralateral S1BF and their amplitude was much smaller than that in the ipsilateral S1BF, thus the power of the 9.5 Hz peak in the contralateral S1BF spectral profile was much smaller than that in the ipsilateral S1BF (see spectral profiles 3 and 4 in Figure 7B).

## Discussion

We induced transient focal brain ischemia by unilateral infusion of Et-1 near the middle cerebral artery. This model offers two advantages: (i) a gradual reperfusion that closely mimics the rate of reperfusion in humans; and (ii) the induction of ischemia without the counfounding effects of anaesthesia. Anaesthesia affects blood pressure, blood gases and body temperature, which might influence stroke severity and outcome, including the mortality rate. This might contribute to explain the lower mortality rate found in both young and middle-aged rats (3.7% and 8.3%, respectively), as compared to values reported in the monofilament or embolic blood clot models of MCA occlusion (6% or 12% for 3-month-old rats, and about 22% and 35% for 12-month and 22-24-month rats) [18,31].

Sensorimotor deficits in ischemic rats were reminiscent of those observed in humans after stroke [32]. One of the most remarkable similarities is the clenching of the affected forepaw fingers and snuggling of this forepaw towards the torso, which we often observed in the PHR test. The behavioral tests used for assessment of asymmetry, i.e., the postural hang reflex, limb placing, limb use asymmetry (cylinder) and adhesive tape removal tests are commonly used for the evaluation of neurological deficits after transient ischemia in rodents [23-27,33-36]. The early PHR test, which we performed as an inclusion/exclusion criterion [23], demonstrated that the Et-1 model could be applied with success to middle-aged rats (excluded only 3 out of 27 ischemic rats) and, therefore, is valuable as a translation model for the evaluation of neuroprotective strategies in human. Most of the studies show that in advanced aged rodents (22-24-months) the outcome of stroke, in terms of infarct size, mortality, behavior, and somatosensory functions, is severely impaired with respect to young rats (References in [2]). However, there are also reports of greater histological damage in young than in old rats, and reduced behavioral deficit in aged rats after embolic stroke (intra-carotid injection of microspheres) [36]. Here we did not find differences in infarct volumes in the two age groups. To our knowledge there are only few studies using middle-aged rats with transient occlusion of MCA [15-18]. Middle-aged (12-month) rats have been shown to acquire stronger neurological motor impairment in beam walking test than young rats (3-month) after photothrombosis of the hindlimb sensorimotor cortex [15]. In another study, robust, reliable and long-term behavioral deficits were found in 16-month-old rats subjected to MCA occlusion, although no comparisons were made with young rats [16]. No differences in ischemic volume and in the percentage of cerebral blood flow reduction in the parietal cortex were found between young and middle aged rats subjected to filament occlusion of MCA [17]. An increase in infarct volume and attenuation of ischemia-induced striatal neurogenesis were reported in 12-month-old rats with filament MCAO as compared to 3-month-old rats [18]. After Et-1 infusion in anaesthetized rats, age was found to have a distinct influence on functional recovery, with 10-days old rats showing a greater potential for plasticity than 6-months old rats [34]. The present study is the only one hitherto that focuses on the middle age period of life (12 month) in freely moving rats subjected to Et-1-induced occlusion of the MCA. We observed greater and longer-lasting asymmetry in ipsilateral-contralateral sensory-motor performance in middle-aged ischemic rats as compared to young ischemic rats for the entire period of observation (14 days after stroke induction). Tests assessing limb use (support on a cylinder and adhesive tape removal) were more sensitive in revealing differences in left-right asymmetry related to aging than the postural hang reflex and limb placing tests. The more pronounced behavioral asymmetry in middle-aged rats with respect to young rats was due to a greater age-dependent impairment of somatosensory functions in the contralateral side of the body in response to ischemia. Having in mind the deleterious impact of age *per se* on the whole organism, a reasonable hypothesis is that old rats (24-27-month old) subjected to ischemia would have worse outcomes than middle-aged rats but this has not been investigated with the Et-1 model as yet. Using the filament occlusion model, comparisons between outputs in old and middle-aged rats are reported in only few studies. In particular, a greater deficit score was reported in 18-month than in 12-month rats, and degenerative morbidity and mortality was shown in 24-month rats [15]. A greater infarct volume in 18-month than in 9-month rats [17], and no difference in infarct volume between 12-month and 18-month rats were also reported [18].

We implemented EEG measurements simultaneously with behavioral observations in an attempt to explain behavioral asymmetry. Only a few studies have examined bioelectrical activity after unilateral infusion of Et-1 in long-term recordings [12,14,20,37]. Here we examined EEG asymmetry by means of the pair-wise Brain Symmetry Index, which measures the asymmetry in power spectral density along homologous cortical channel pairs of both hemispheres [29,38]. These studies showed that pdBSI may be of value in assisting visual interpretation of EEG during carotid endarterectomies, in correlating clinical severity in patients with ischemic cerebrovascular disease (transitory ischemic attacks, stroke), and in monitoring patients with anterior circulation syndrome of presumed ischemic origin. Importantly, the degree of EEG asymmetry of the sensorimotor cortex between lesioned and non-lesioned corticospinal systems at long times after the onset of stroke in humans is inversely related to recovery of motor function [39]. This strengthens the importance of mechanisms of homeostatic plasticity during recovery from focal, unilateral stroke. Analysis of the interhemispheric EEG asymmetry in models of focal ischemia in rodents has not been carried out, as yet. Recently, the BSI index approach was used in swines to show the utility of quantitative EEG for diagnosis and prognosis of cerebral arterial gas embolism [40]. In their short-term EEG investigation after embolization (240 min), the authors have shown that temporal BSI correlated with intracranial pressure, brain lactate and brain oxygen tension. In the present study, we showed that pdBSI in young rats increased shortly after Et-1 (at 15 and 60 min) compared with recordings before Et-1, while in aged rats pdBSI after ischemia was greater than pdBSI before ischemia or pdBSI in young rats during the whole experimental period (up to 14 days post-Et-1). These finding suggest that middle-aged rats were more vulnerable to Et-1-induced ischemia than young rats.

The regional analysis of spectral density EEG profiles showed in both aged groups a suppression of the EEG power (except for the slowest frequencies) in the ipsilateral somatosensory areas during the acute period of ischemia, which paralleled the behavioral deficits. A relationship between reduction of fast-band EEG power in the affected hemisphere and impairment of hand functionality was reported in humans [41]. The increase in the EEG power of the slow delta wave components in ipsilateral somatosensory cortex at H4 and again at D3 coincided with the periods of reperfusion and established brain damage during the chronic phase, respectively [11]. The alterations in the oscillating cortical slow activity in the ipsilateral somatosensory cortex during and after the reperfusion phase of Et-1-induced ischemia might be due to reoxygenation of the ischemic tissue, which is known to provide chemical substrates for intracellular mechanisms leading to neuronal death and neurological deficits [42]. Oxidative stress-related generation of toxic compounds, such as free radicals, reactive oxygen or nitrogen species during the reperfusion phase [43] may be detrimental to neurons, thereby causing EEG abnormalities. A close association between EEG/MEG abnormalities and some markers of the oxidative stress or free radical scavengers (iron, peroxides and transferrin) have been found in patients with Alzheimer disease [44] and stroke [45]. It will be a challenge to trace a path from the molecular events associated with oxidative stress during ischemia to the excitability of the cerebral cortex at the EEG level after stroke in experimental animals and humans.

We also found changes in the EEG power distribution in the contralateral somatosensory cortical areas, which were opposite to those seen in the ipsilateral areas in the ischemic middle-aged rats: while in the ipsilateral cortex the EEG was suppressed during the acute phase of ischemia, in the contralateral cortex the EEG power was increased. This is in line with data obtained in rodents [46] and humans [47] after stroke. Numerous studies have examined the role of the contralateral cortex in functional recovery after unilateral stroke. Contralateral synchronized neuronal activity occurs as a remote effect of the primary ischemic lesion (the diaschisis phenomenon) one to three days after stroke in experimental animals [48]. In humans, ischemia-induced neuronal reorganization, which extends to the contralateral areas, is supposed to be associated with interhemispheric asymmetry of inhibition [49]. Neuronal sprouting, formation of new synapses and increased dendritic arborization of pyramidal neurons occurring in the contralateral hemisphere are associated with the recovery of sensorimotor function following unilateral stroke in rats [50]. Autoradiographic data show a role for the contralateral cortex in the behavioral outcome and maintenance of the recovered state of ischemic rats with unilateral filament MCA occlusion [33]. PET and fMRI studies in rats show increased blood flow and metabolic activity in the contralateral hemisphere, which parallels functional recovery after stroke [51]. The poor functional recovery of aged rats after stroke has been related to a reduced transcriptional activity in the contralateral hemisphere [52]. Contrast-enhanced fMRI also showed the involvement of the contralateral somatosensory fields in brain reorganization in rats subjected to unilateral MCA occlusion [53]. EEG power increase in the contralateral cortex in patients with unilateral ischemic stroke in the MCA territory was associated with negative prognosis of stroke [54].

An interesting finding was the observation of appearance of HVSs in the contralateral S1BF in the middle-aged rats by days 7–14 after stroke induction. HVSs are generalized EEG patterns typically seen in rats older than 6–8 months that are motionless during waking state [30,55] and are thought to represent a distinct functional mode of thalamo-cortical circuits [56]. The role of the thalamus in the recovery processes after ischemia is not clear. Data on changes in thalamus after unilateral ischemia or damage in the cortex are scare in preclinical models of stroke. Interestingly, two weeks after occlusion of MCA, the fMRI response to stimulation of the affected forelimb is shown to be absent in the ipsilateral thalamus although it can be detected in the ipsilesional cortex [57]. Changes in neuroplasticity occurs in the thalamus, where retrograde neuronal degeneration has been found after cortical damage [58]. However, to our knowledge there are no studies that examine how the age- and motionless-related rhythmical patterns like HVSs might be influenced by stroke. In our longitudinal EEG studies we observed high expression of HVSs in ipsilateral cortex one to eight months after Et-1 infusion. These findings are in line with reported enhanced rhythmogenic properties of the ipsilateral thalamocortical neurons with long delay (days, months) after photothrombotic focal cortical stroke in 7-8-month old rats [59]. The functional significance of HVSs during quiet waking in rodents is not clear, as yet [60] and it is not known how they might influence the recovery process after stroke. Analyzing together the EEG data obtained from middle-aged rats monitored for 14 days after Et-1 infusion and EEG data from rats monitored for 8 months after Et-1 infusion, we showed that HVSs appeared first in the contralateral cortex (7–14 days) and later (after the first month) in the ipsilateral cortex. These findings lend credit to the hypothesis of the biphasic sequence of events after occlusion of MCA in rats (fMRI experiments): an initial reorganization in the contralesional hemisphere and a late recruitment of the ipsilesional periinfarct cortex [53]. The roles of contralateral and ipsilesional cortical areas together with the thalamocortical circuits in the recovery process and plasticity after unilateral stroke-induced ischemia merit further investigation.

## Conclusions

In the present study, we have used a multiparametric approach to validate on middle-aged rats the widely used endothelin-1 model of unilateral stroke carried-out usually in young rats. The middle-aged rats subjected to endothelin-1-induced ischemia showed robust behavioral and EEG changes, which were more severe and longer lasting than in young rats. The mechanisms underlying the response of the aged brain to ischemia are still unclear but the present data show that this response may affect the degree and rate of recovery. This model is potentially translational because middle-aged rats match the human population at risk according to the recent reports for rejuvenation of stroke. Our results suggest that stroke outcome may be worse earlier in life than in late senescence. The serial multifunctional assessment of various behavioral and electrophysiological outputs over a long time frame performed in the present study may serve as clinically-relevant experimental design for investigation of processes of recovery, brain reorganization and adaptive plasticity after stroke. A better knowledge of alterations in stroke outcomes in middle-aged rats has important preventive and management implications providing clues for future investigations on effects of various neuroprotective and neurorestorative drugs against cerebrovascular accidents that may occur before late senescence.

## Competing interests

The authors declare that they have no competing interests.

## Authors’ contributions

SM designed and conceptualized the study, participated in all phases of the experiments, analyzed and interpreted the data, carried out the statistical analysis and figures, and drafted and finalized the manuscript. RM participated in the experiments, analysis of behavioral data and the spectral analysis of the EEG data. LK participated in the experiments, carried out the spectral analysis of the EEG data, made one of the figures. FN and RN contributed to writing and revision of the manuscript. All authors read and approved the final version of the manuscript.
